# Validity and test-retest reliability of the self-completion adult social care outcomes toolkit (ASCOT-SCT4) with adults with long-term physical, sensory and mental health conditions in England

**DOI:** 10.1186/s12955-017-0739-0

**Published:** 2017-08-18

**Authors:** Stacey Rand, Juliette Malley, Ann-Marie Towers, Ann Netten, Julien Forder

**Affiliations:** 1 Quality and Outcomes of person-centred care policy Research Unit (QORU) and Personal Social Services Research Unit (PSSRU), Cornwallis Building, University of Kent, Canterbury, CT2 7NF UK; 2 Quality and Outcomes of person-centred care policy Research Unit (QORU) and Personal Social Services Research Unit (PSSRU), Cowdray House, London School of Economics and Political Science, Houghton Street, London, WC2A 2AE UK

**Keywords:** Scale development/validation, Quality of care, Long-term care, Ascot

## Abstract

**Background:**

The Adult Social Care Outcomes Toolkit (ASCOT-SCT4) is a multi-attribute utility index designed for the evaluation of long-term social care services. The measure comprises eight attributes that capture aspects of social care-related quality of life. The instrument has previously been validated with a sample of older adults who used home care services in England. This paper aims to demonstrate the instrument’s test-retest reliability and provide evidence for its validity in a diverse sample of adults who use publicly-funded, community-based social care in England.

**Methods:**

A survey of 770 social care service users was conducted in England. A subsample of 100 services users participated in a follow-up interview between 7 and 21 days after baseline. Spearman rank correlation coefficients between the ASCOT-SCT4 index score and the EQ-5D-3 L, the ICECAP-A or ICECAP-O and overall quality of life were used to assess convergent validity. Data on variables hypothesised to be related to the ASCOT-SCT4 index score, as well as rating of individual attributes, were also collected. Hypothesised relationships were tested using one-way ANOVA or Fisher’s exact test. Test-retest reliability was assessed using the intra-class correlation coefficient for the ASCOT-SCT4 index score at baseline and follow-up.

**Results:**

There were moderate to strong correlations between the ASCOT-SCT4 index and EQ-5D-3 L, the ICECAP-A or ICECAP-O, and overall quality of life (all correlations ≥ 0.3). The construct validity was further supported by statistically significant hypothesised relationships between the ASCOT-SCT4 index and individual characteristics in univariate and multivariate analysis. There was also further evidence for the construct validity for the revised *Food and drink* and *Dignity* items. The test-retest reliability was considered to be good (ICC = 0.783; 95% CI: 0.678–0.857).

**Conclusions:**

The ASCOT-SCT4 index has good test-retest reliability for adults with physical or sensory disabilities who use social care services. The index score and the attributes appear to be valid for adults receiving social care for support reasons connected to underlying mental health problems, and physical or sensory disabilities. Further reliability testing with a wider sample of social care users is warranted, as is further exploration of the relationship between the ASCOT-SCT4, ICECAP-A/O and EQ-5D-3 L indices.

## Background

Long-term care (also referred to as social care in the UK) covers a range of services designed to enable people with physical, intellectual, psychological or ageing-related support needs to maintain their independence and quality of life. In the UK, social care services include various community- based services (e.g. home care, day centres, meals services, equipment, home adaptations or assistance aids and professional support from a care manager or social worker), as well as residential care in institutional settings. Social care systems across Europe vary in their balance of informal or unpaid care and formal care provided by social care services; however, ageing populations are projected to increase overall demand for formal care in Europe over the next few decades [1]. In this context, and alongside a reduction in public spending on social care in some European countries [2], there is heightened interest in how to evaluate the (cost-)effectiveness of social care services to make the best use of resources.

The evaluation of the effectiveness of social care requires measurement instruments that are designed to capture the objectives of social care. Although social care interventions address the functional impairments of people with social care needs (e.g. the ability to wash or dress oneself), the ultimate aim of social care is to compensate for the effect of impairments on quality of life [3]. Long-term social care interventions do not directly seek to improve health status but respond to fluctuations or decline in health in order to improve or maintain quality of life over time [3]. It has been increasingly recognised that measures of health-related quality of life do not capture all of the relevant aspects of quality of life valued by service users and are not adequately sensitive to the effects of social care interventions on quality of life [4–8]. The evaluation of social care requires instruments that capture the compensatory effect of services on valued aspects of quality of life, rather than measures limited only to functional ability or health-related quality of life. The Adult Social Care Outcomes Toolkit (ASCOT) is a suite of instruments designed to capture social care-related quality of life (SCRQoL), which is defined as aspects of quality of life that are important to social care service users and may be compensated for by social care support [9].

One of the ASCOT instruments, the ASCOT-SCT4, was developed as a self-report instrument for the evaluation of a diverse range of social care interventions and is included in the Adult Social Care Outcomes Framework (ASCOF) as an overarching indicator of social care outcomes to inform policy, planning and administration by local and national government [10]. The ASCOT-SCT4 is a multi-attribute utility index. Each of the eight attributes (see Table 1) is captured by a single item with four response level*.* The highest level captures the concept of ‘capability’ [11, 12] while the other three response options relate to states of ‘functioning’ [13]. The instrument was developed in an iterative manner, drawing on expert review and the views of adults who use social care services to identify relevant quality of life attributes. In particular, it drew on a five-year programme of work that examined how adults who use social care services and their carers define social care outcomes [14–17]. The attributes were also reviewed in the context of the literature and analysis of early versions of the measure [13]. The questions were refined through 30 cognitive interviews with adults who had mental and/or physical long-term health conditions [13]. The ASCOT-SCT4 utility index is calculated by applying weights derived from a combined best-worst scaling (BWS) and time-trade-off (TTO) model [13, 18, 19]. The ASCOT index can therefore be used to calculate quality-adjusted life years (QALYs) and value quality of life gains from social care interventions.

**Table 1:** ASCOT-SCT4 attributes

Attribute	Definition
Control over daily life	The respondent is able to choose what to do and when to do it, having control over daily life and activities
Personal cleanliness and comfort	The respondent feels personally clean and comfortable and looks presentable. At best, is dressed and groomed in a way that reflects personal preferences
Food and drink	The respondent feels that s/he has a nutritious, varied and culturally appropriate diet with enough food and drink, at regular and timely intervals, that he/she enjoys
Personal safety	The respondent feels safe and secure. This means being free from fear of abuse, falling or other physical harm and fear of being attacked or robbed
Social participation and involvement	The respondent feels content with his/her social situation, where social situation is taken to mean the sustenance of meaningful relationships with friends and family, and feeling involved or part of a community, should this be important to the service user
Occupation	The respondent is sufficiently occupied in a range of meaningful activities whether it be formal employment, unpaid work, caring for others or leisure activities
Accommodation cleanliness and comfort	The respondent feels that the home environment, including all rooms, is clean and comfortable
Dignity	The psychological impact of the way support and care services are provided on the service user’s personal sense of significance and sense-of-self

The ASCOT-SCT4 has been recommended as an instrument for the evaluation of social care for older adults, but it is recognised that further validation of the instrument with a wider range of users of adult social care services would be valuable [7, 20]. The construct validity of the instrument (that is, the extent to which it captures the measurement construct of social care-related quality of life) has been evaluated using data collected from a survey of 301 older adults in England who used home care services [9, 13]. The validity of the ASCOT-SCT4 index was evaluated by comparing the ASCOT-SCT4 index to instruments that captured the theoretically-related constructs of health-related quality of life and also control and autonomy [9, 13], and further analysis focussed on the response properties and construct validity of the individual ASCOT-SCT4 attributes [9]. Other studies to explore the content validity of the instrument and its convergent validity in community-dwelling older adults have been conducted in Australia [6, 20] and in the Netherlands using a culturally- and linguistically-validated Dutch translation of the instrument [8, 21]. There is, as yet, however, limited evidence of the validity of the instrument in younger adults, aged 18 to 64 years, or in samples of service users with social care support needs related to mental health conditions. Furthermore, the test-retest reliability of the instrument has only been established for the Dutch translation of ASCOT-SCT4 [8].

The aim of this article is to explore the construct validity, feasibility and test-retest reliability of the English version of the ASCOT-SCT4 instrument. In terms of construct validity, we focus on the ability of the measure to capture aspects of quality of life that are relevant to social care users. We also examine its performance in relation to instruments capturing conceptually-related constructs that are hypothesised to be related to social care-related quality of life. This paper therefore expands upon the study reported by Netten et al. [13] and Malley et al. [9], which provided support for the validity of ASCOT-SCT4 in a sample of home care service users, aged 65 and over. In this paper, we examine the construct validity of the measure with a diverse sample of social care service users, including adults aged 18 to 64 years and people with mental health conditions, and the test-retest reliability with a subgroup of adults with physical and sensory disabilities.

## Methods

### Design and setting

A survey of community-based adult social care service users was conducted across 22 local authorities (LAs) in England. LA adult social services departments and home care providers identified the sample from social care records based on the following eligibility criteria: aged 18 years or over, in receipt of fully or partly publicly-funded community-based social care services, not in residential or nursing care, and primary support reason recorded as physical disability or sensory impairment (PDSI) or mental health condition (MH) or learning disability (LD).

Follow-up interviews were completed for a subsample of the respondents with a primary support reason of physical disability or sensory impairment (*n* = 100). The analyses presented in this article exclude the 17 cases where the follow-up interview was erroneously completed within 7 days or after 21 days of the baseline interview.

Data were collected by face-to-face or by telephone computer-aided interview conducted between June 2013 and March 2014. Written or verbal consent was obtained before all interviews.

Ethical approval for the study was given by the national social care research ethics committee in England (12/IEC08/0049).

### Questionnaire

The questionnaire included the version of the ASCOT-SCT4 recommended by Netten et al. [13] with two exceptions. As Netten et al. relate, the highly skewed distribution of responses to the items for *Food and drink* and *Accommodation* in an earlier version of the ASCOT-SCT4 led to proposed revisions to these items [13]. In this study, the revised *Food and drink* item was included; however, the revised *Accommodation* item was erroneously omitted. Second, the evaluation of the construct validity of the *Dignity* item with older adults in England identified some unexpected associations [9], which led to the revision of the *Dignity* item in this study to refer only to care provided by social care services rather than also include family or friend carers (see Appendix). When scored with the utility weights, the ASCOT-SCT4 is a continuous scale from −0.17 to 1.0 (full social care-related quality of life, SCRQoL) with zero equivalent to ‘being dead’ [13]. As with other measures that can be used to calculate QALYs, values less than zero represent SCRQoL states that are considered worse than being dead.

The questionnaire also included the EuroQOL-5D (EQ-5D-3 L), which was scored with UK preference values (UK TTO) [22–24]. The EQ-5D-3 L is a five-item instrument (mobility, self-care, usual activities, pain/discomfort, and anxiety/depression) with three response options (no problems, some problems, and extreme problems). The utility-weighted index score ranges from −0.594 (extreme problems on all five items) to 1.0 (full health) with zero equivalent to ‘being dead’. The Investigating Choice Experiments for the Preferences of Older People Capability measure for adults (ICECAP) measures for adults aged 18–64 years (ICECAP-A) or older adults aged 65 or over (ICECAP-O) were also included in the questionnaire. These five-item instruments capture the individual’s ability to ‘do’ and ‘be’ the things that are important to them in life (‘capability wellbeing’) [25–28]. The five items in the ICECAP-A are attachment, stability, achievement, enjoyment and autonomy, whereas the ICECAP-O includes attachment, security, role, enjoyment and control. Each item has four response levels that describe capability in each attribute as: none, a little, a lot, and all. The UK preference-weights were applied to convert the capability states into a scale from 0 (no capability) to 1 (full capability) [26, 29].

The baseline interviews also collected socio-demographic characteristics. Items on thirteen activities of daily living (ADLs) and instrumental activities of daily living (IADLs) from the 65+ Social Care questionnaire were used to assess social care need [30]. Overall quality of life was measured using a self-rated seven-point scale. Individual perceptions of social isolation were measured using the UCLA Three-Item Loneliness Scale [31]. Two items from the Adult Social Care Survey in England were also included to measure the individual’s rating of the suitability of their home environment and accessibility of the local area outside of the home [32, 33]. All respondents were asked to rate their satisfaction with social care services on a seven-point scale from extremely satisfied to extremely dissatisfied. The adults who received home care services were asked a series of questions to capture aspects of service quality [32, 34]: whether care workers arrive at suitable times, on time and not in a rush; whether they are always kept informed of changes to the home care service; whether care workers do what the respondent wants done; whether the carer workers spend less time per visit than they are supposed to; whether the respondent is happy with how care workers treat him/her (dignity); and the quality of the relationship between the care worker and respondent.

The follow-up interviews included the ASCOT-SCT4, the thirteen I/ADLs, and two items to capture any self-reported change in overall health or quality of life since the baseline interview.

### Analysis

Analyses were conducted in Stata version 13 [35]. The analysis excludes cases where all items in the ASCOT-SCT4 were answered by someone else without consultation with the service user (*n* = 22).

#### Feasibility

The feasibility of the ASCOT-SCT4 was evaluated by the percentage of missing values for each item.

#### Construct validity

Since there is no alternative ‘gold standard’ instrument for the measurement construct of social care-related quality of life, here we evaluate the construct validity of the ASCOT-SCT4 index by examining its relationship with other measures of similar or related constructs (convergent validity). Convergent validity was evaluated using the Spearman Rank Correlation Coefficient between the utility-weighted ASCOT-SCT4 score and three measures of constructs theoretically related to social care-related quality of life: health-related quality of life (EQ-5D-3 L); capability wellbeing (ICECAP-A/O); and overall quality of life rated on a single-item seven-point scale. Correlation coefficients were interpreted as weak (<0.3), moderate (≥0.3, <0.5) or strong (>0.5) [36].

Since the ASCOT-SCT4 is designed to capture aspects of quality of life broader than health, as is also the ICECAP-A/O and the overall quality of life item, the ASCOT-SCT4 index was expected to be less strongly correlated to the EQ-5D-3 L than the ICECAP-A/O [8]. Because the fourth level of response for the ASCOT-SCT4 relates to a level of social care need that may affect health, however, we anticipated a weak-moderate relationship between the ASCOT-SCT4 and EQ-5D-3 L. The ASCOT-SCT4 and ICECAP instruments were anticipated to be more strongly correlated than either instrument with EQ-5D-3 L because the underlying constructs of SCRQoL (ASCOT) and capability wellbeing (ICECAP) share a common focus on aspects of wellbeing or quality of life beyond health. This analysis was conducted for the whole sample and also for three subgroups: (1) adults with mental health conditions; (2) adults with physical disability/sensory impairment aged between 18 and 64 years; and (3) adults with physical disability/sensory impairment aged 65 years or older.

Construct validity may also be explored through the extent to which the measure relates to contextual variables hypothesised to be associated with the measurement construct [37]. In this study, the construct validity of ASCOT was also assessed by testing the hypothesised relationships between individual characteristics and ASCOT-SCT4 (see Table 2). These hypothesised relationships were tested using one-way analysis of variance (ANOVA) of the whole sample and also the three subgroups. The construct validity of the two revised items (*Dignity*, *Food and drink*) was also evaluated by testing associations between these items and characteristics hypothesised to be related to them. Associations were tested using Fisher’s exact test due to instances where the expected frequencies in each cell were fewer than five.

**Table 2:** Expected associations with ASCOT-SCT4 index or the revised items (*Food and drink*, *Dignity*)

Variable	Expected Associations
Health and disability	
ADLs and IADLs	ADLs and IADLs capture how well an individual is able to undertake everyday activities without the compensatory action of social care support. As such, I/ADLs are often used as measures of ‘need’ in social care research. Since the ASCOT-SCT4 response options relate to ‘needs’, as well as preferences and choice (i.e. ‘ideal state’), it is anticipated that difficulty undertaking I/ADLs will be negatively associated with the ASCOT-SCT4 index. The food-related I/ADLs (i.e. feed self and shopping) are also expected to be negatively associated with *Food and drink*.
Home and local environment	
Self-rated suitability of home design	Poor rating of the design of the home in relation to an individual’s needs may make it more difficult to provide safe and optimal care in the home environment. Therefore, a positive relationship between rating of more suitable home design and ASCOT-SCT4 index score was expected.
Accessibility of the local area	We expected poor accessibility of the local area to be associated with poorer social care-related quality of life due to limitations it places on ability to get around outdoors for leisure or social activities or feeling able to make choices about what to do and where to go.
Social contact and loneliness	
Three-item Loneliness Scale	A negative association between increased loneliness and ASCOT-SCT4 index score was expected due to the relationship between loneliness, depression and quality of life [38].
Service satisfaction and quality	
Quality of home care	A positive association was expected between these items capturing aspects associated with the quality of the delivery of care by care workers (e.g. care workers come at suitable times, do things you want done, arrive on time, not in a rush or spend less time than supposed to, and respondent is kept informed about changes in care) and *Dignity*. In particular, we expected the global rating of the way the person felt they were treated by the care worker to be associated with *Dignity.*
Satisfaction with services	A positive association was expected between satisfaction with social care services and social care-related quality of life, for the ASCOT-SCT4 index score and also the item ratings for *Dignity* and *Food and drink*.

Whilst controlling for other factors, multivariate regression was used to test the hypothesised relationships and also for differences by primary support reason, age group and survey administration by face-to-face or telephone interview. An ordinary least squares (OLS) regression was calculated with the hypothesised factors in Table 2 as the independent variables and the ASCOT-SCT4 index as the dependent variable. To test any systematic differences between subgroups, whilst controlling for other factors hypothesised to be related to SCRQoL, the respondent’s client group (physical disability/sensory impairment; mental health) and age group (18–64 years; ≥65 years) were also considered as independent variables. The method of administration was also included as a dummy variable in the model to consider potential bias associated with the administration of the survey by face-to-face or telephone interview.

#### Test-retest reliability

The intra-class correlation coefficient (ICC) is used to assess the test-retest reliability of the ASCOT-SCT4 index [38, 39], and the quadratic weighted kappa statistic (κ) is used for each individual attribute [40]. Adequate test-retest reliability was defined as ICC ≥0.75 [41], and the κ values were defined as poor to fair (0.00–0.40), moderate (0.41–0.60), substantial (0.61–0.80) to almost perfect (0.81–1.00) [40]. Since κ may be low if the number of observations is small [42], percentage agreement between time-points was also calculated with an agreement of ≥66% classified as adequate [43].

## Results

A total of 770 interviews were completed, of which 748 cases were considered in the analyses due to the exclusion of instances where the ASCOT-SCT4 was completed by proxy (*n* = 22). The characteristics of the sample considered in the analysis are presented in Table 3. The study sample has a lower proportion of older adults (52.8%) compared to the representative national sample of people who receive publicly-funded social care in the English ASCS (68.5%) [44]; however, this difference is likely to reflect the oversampling in this study of adults with mental health conditions, who tend to be younger, to enable a separate analysis of this subgroup. The proportion of females in the study sample is comparable to the ASCS (58.3% and 62.8% respectively) [44].

**Table 3:** Sample characteristics

	Overall(*n* = 748)	Follow-up (*n* = 83)	Mental Health (*n* = 214)	Physical Disability or Sensory Impairment18–64 years (*n* = 197)	Physical Disability or Sensory Impairment≥65 years (*n* = 337)
	Freq. (%)	Freq. (%)	Freq. (%)	Freq. (%)	Freq. (%)
Sex					
Female	436 (58.3%)	48 (57.8%)	115 (53.8%)	108 (54.8%)	213 (63.2%)
Male	312 (41.7%)	35 (42.2%)	99 (46.2%)	89 (45.2%)	124 (36.8%)
Age					
18–64 years	353 (47.2%)	39 (47.0%)	156 (72.9%)	n/a	n/a
65+ years	395 (52.8%)	44 (53.0%)	58 (27.1%)	n/a	n/a
I/ADLs with difficulty^a^					
None	38 (5.1%)	0 (0.0%)	24 (11.2%)	4 (2.0%)	10 (3.0%)
1–4	111 (14.8%)	9 (10.8%)	69 (32.2%)	21 (10.7%)	21 (6.2%)
5–8	182 (24.3%)	18 (21.7%)	52 (24.3%)	32 (16.2%)	98 (29.1%)
9–12	225 (30.1%)	32 (38.6%)	38 (17.8%)	74 (37.6%)	113 (33.5%)
13	128 (17.1%)	14 (16.8%)	12 (5.6%)	51 (25.9%)	65 (19.3%)
Missing	64 (8.6%)	10 (12.1%)	19 (8.9%)	15 (7.6%)	30 (8.9%)
Interview mode of administration					
Face-to-face	557 (74.5%)	45 (54.2%)	160 (74.8%)	139 (70.6%)	258 (76.6%)
Telephone	191 (25.5%)	38 (45.8%)	54 (25.2%)	58 (29.4%)	79 (23.4%)
Change in self-rated health from baseline to follow-up					
Worse health	n/a	19 (22.9%)	n/a	n/a	n/a
No change	n/a	45 (54.2%)	n/a	n/a	n/a
Better health	n/a	18 (21.7%)	n/a	n/a	n/a
Missing	n/a	1 (1.2%)	n/a	n/a	n/a
Change in I/ADLs from baseline to follow-up					
Fewer I/ADLs with difficulty at follow-up	n/a	12 (14.4%)	n/a	n/a	n/a
No change between baseline and follow-up	n/a	22 (26.5%)	n/a	n/a	n/a
More I/ADLs with difficulty at follow-up	n/a	33 (39.8%)	n/a	n/a	n/a
Missing	n/a	16 (19.3%)	n/a	n/a	n/a
Change in overall QoL from baseline to follow-up					
Better QoL	n/a	8 (9.6%)	n/a	n/a	n/a
Much the same	n/a	63 (75.9%)	n/a	n/a	n/a
Worse QoL	n/a	12 (14.5%)	n/a	n/a	n/a
	Mean (SD, N)	Mean (SD, N)	Mean (SD, N)	Mean (SD, N)	Mean (SD, N)
ASCOT-SCT4 Index (baseline)	0.732 (0.209, 719)	0.734 (0.239, 78)	0.716 (0.214, 205)	0.693 (0.232, 191)	0.765 (0.185, 323)
ASCOT-SCT4 Index (follow-up)	n/a	0.730 (0.233, 79)	n/a	n/a	n/a
EQ-5D-3 L Index (baseline)	0.277 (0.392, 741)	0.176 (0.356, 81)	0.392 (0.410, 213)	0.148 (0.365, 193)	0.278 (0.374, 335)
ICECAP-A Index (baseline) ^b^	0.599 (0.233, 249)	0.604 (0.210, 19)	0.608 (0.232, 112)	0.591 (0.233, 137)	n/a
ICECAP-O Index (baseline) ^b^	0.686 (0.223, 291)	0.717 (0.192, 26)	0.657 (0.238, 44)	n/a	0.691 (0.220, 247)

^a^ The 13 I/ADLs included in the interview are: getting in and out of bed; washing hands and face; having a bath or shower; dressing or undressing; using the toilet; eating (including cutting up food); taking medicines; getting around indoors; getting up or down stairs; getting out of the house; shopping; routine housework or laundry; and paperwork or paying bills

^b^ The ICECAP-O and ICECAP-A were not included when the questionnaire was administered by telephone to reduce the length/duration of the interviews

**Fig. 1:**
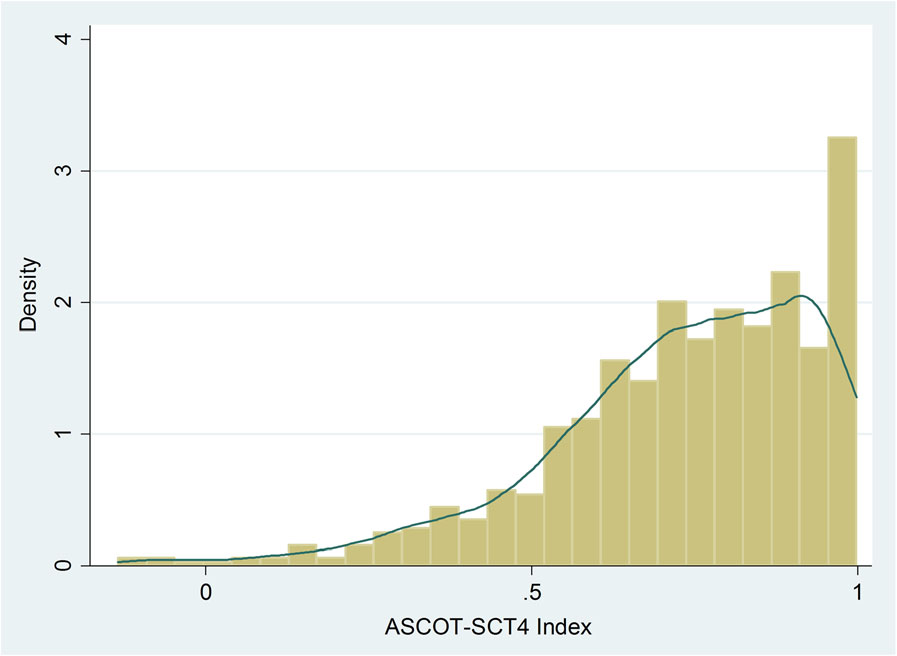
Distribution of ASCOT-SCT4 Index scores

The ASCOT-SCT4 Index was negatively skewed with a possible ceiling effect at the upper end of the scale (see Fig. 1). The rate of missing data for the ASCOT-SCT4 items less than 1%, except for *Dignity* (3.3%).^1^ This suggests that the items were acceptable to respondents. The distribution of responses at the ‘ideal state’ range for each item from 25.1% (*Occupation*) to 69.8% (*Food and drink*). Correspondingly, only 6.9% of the sample reported some or high-level needs for *Food and drink.*

### Construct validity

Correlations between the ASCOT-SCT4 Index and the EQ-5D-3 L, ICECAP-A/O and overall QoL are shown in Table 4. There was a moderate positive association between the ASCOT-SCT4 and overall EQ-5D-3 L index. The EQ-5D’s mobility, self-care and pain/discomfort items were weakly correlated with the ASCOT-SCT4 with stronger, moderate correlations with anxiety/depression and usual activities. This may reflect the expected relationships between social care need, which may be partially captured by the EQ-5D-3 L usual activities item, as well as anxiety or depression, and social care-related quality of life. As hypothesised, the positive correlations between SCRQoL and overall QoL were strong in the overall sample and subgroup analyses (ρ > 0.50), and the strongest associations were found between ASCOT-SCT4 score and ICECAP-A (ρ >0.62) and ICECAP-O (ρ >0.64) in the analysis of the whole sample and also in each of the three subgroups.

**Table 4:** Spearman’s rank correlation coefficients between the ASCOT-SCT4 Index and EQ-5-3 L, ICECAP-O/A and overall QoL

	Overall *(N)*	Mental Health *(N)*	Physical Disability or Sensory Impairment18–64 years *(N)*	Physical Disability or Sensory Impairment≥65 years *(N)*
EQ-5D	0.370** (712)	0.472** (204)	0.327** (187)	0.333** (321)
*Mobility*	0.150* (716)	0.266* (205)	0.060 (189)	0.166 (322)
*Self-care*	0.188** (719)	0.250 (205)	0.123 (191)	0.220** (323)
*Usual activities*	0.337** (718)	0.441** (205)	0.316** (190)	0.344** (323)
*Pain/discomfort*	0.248** (718)	0.313** (205)	0.320** (190)	0.159 (323)
*Anxiety/depression*	0.360** (717)	0.396** (204)	0.312** (191)	0.330** (322)
Overall QoL	0.552** (716)	0.510** (205)	0.601** (190)	0.551** (321)
ICECAP-O ^a^	0.670** (280)	0.767** (42)	n/a	0.645** (238)
*Attachment*	0.366** (287)	0.299 (43)	n/a	0.379** (244)
*Security*	0.434** (285)	0.518 (42)	n/a	0.419** (243)
*Role*	0.563** (289)	0.612* (44)	n/a	0.552** (245)
*Enjoyment*	0.632** (288)	0.741** (44)	n/a	0.607** (244)
*Control*	0.532** (289)	0.614** (44)	n/a	0.508** (245)
ICECAP-A ^a^	0.624** (243)	0.625** (109)	0.623** (134)	n/a
*Stability*	0.521** (245)	0.548** (110)	0.502** (135)	n/a
*Attachment*	0.363** (245)	0.382** (110)	0.366** (135)	n/a
*Autonomy*	0.334** (245)	0.349* (110)	0.301* (135)	n/a
*Achievement*	0.534** (244)	0.486** (110)	0.564** (134)	n/a
*Enjoyment*	0.549** (244)	0.511** (109)	0.583** (135)	n/a

*p < 0.05, **p < 0.01

^a^ The ICECAP-O and -A were not included in the telephone interviews to reduce the length/duration of the questionnaire

Table 5 presents the univariate analyses to test the hypothesised associations between ASCOT-SCT4 and individual characteristics, which are outlined in Table 2. All of the hypothesised relationships reached statistical significance at the 5% level in the analysis of the whole sample (*n* = 748) and the subsample of adults with PDSI aged 65 years or older (*n* = 337). Post-hoc tests with Bonferroni correction confirmed a statistically significant between-group difference for all comparisons. In the analysis by the other subgroups, five of the hypothesised relationships did not reach significance for adults with MH conditions (*n* = 214) and three of the hypothesised relationships did not reach significance for younger adults with PDSI (*n* = 197); however, this may be partially explained by a loss in statistical power due to the small numbers in some categories.

**Table 5:** ASCOT-SCT4 index score by individual characteristics

	Overall (*n* = 748)		Mental health (*n* = 214)		Physical Disability or Sensory Impairment 18–64 years (*n* = 197)		Physical Disability or Sensory Impairment 65+ years (*n* = 337)	
	SCRQoL Mean (N)	F Statistic	SCRQoL Mean (N)	F Statistic	SCRQoL Mean (N)	F Statistic	SCRQoL Mean (N)	F Statistic
*Health and disability*								
*Difficulty: In/out bed*		21.10***		8.92**		3.99*		10.69**
No	0.77 (328)		0.75 (139)		0.75 (52)		0.80 (137)	
Yes	0.70 (391)		0.65 (66)		0.67 (139)		0.74 (186)	
*Difficulty: Wash hands & face*		25.69***		1.86		4.90*		27.02***
No	0.76 (478)		0.73 (172)		0.73 (101)		0.80 (205)	
Yes	0.68 (241)		0.67 (33)		0.65 (90)		0.70 (118)	
*Difficulty: Bath or shower*		26.47***		4.54*		17.13***		17.43***
No	0.79 (219)		0.75 (111)		0.83 (37)		0.84 (71)	
Yes	0.71 (498)		0.68 (94)		0.66 (154)		0.74 (250)	
*Difficulty: Dressing*		21.01***		10.32**		10.07**		11.18***
No	0.78 (227)		0.76 (121)		0.79 (42)		0.83 (64)	
Yes	0.71 (492)		0.66 (84)		0.66 (149)		0.75 (259)	
*Difficulty: Using toilet*		26.17***		6.35*		5.61*		17.53***
No	0.77 (406)		0.74 (157)		0.74 (76)		0.80 (173)	
Yes	0.69 (313)		0.65 (48)		0.66 (115)		0.72 (150)	
*Difficulty: Eating*		34.02***		13.70***		6.48*		18.39***
No	0.77 (384)		0.75 (141)		0.74 (82)		0.81 (161)	
Yes	0.68 (335)		0.64 (64)		0.66 (109)		0.72 (162)	
*Difficulty: Taking medicine*		17.24***		2.97^		1.86		15.14***
No	0.76 (382)		0.74 (114)		0.71 (93)		0.80 (175)	
Yes	0.70 (331)		0.69 (91)		0.67 (96)		0.72 (144)	
*Difficulty: Get around indoors*		28.91***		10.78**		11.54***		23.05***
No	0.78 (276)		0.75 (145)		0.79 (49)		0.85 (82)	
Yes	0.70 (442)		0.64 (60)		0.66 (142)		0.74 (240)	
*Difficulty: Up/down stairs*		9.10**		3.32^		6.28*		8.09**
No	0.77 (153)		0.74 (102)		0.81 (19)		0.85 (32)	
Yes	0.72 (511)		0.69 (86)		0.67 (159)		0.75 (266)	
*Difficulty: Get out of house*		21.14***		5.79*		9.11**		14.99***
No	0.80 (145)		0.76 (76)		0.81 (27)		0.87 (42)	
Yes	0.71 (574)		0.69 (129)		0.67 (164)		0.75 (281)	
*Difficulty: Shopping*		7.32**		3.80^		2.76^		4.85*
No	0.78 (103)		0.76 (62)		0.78 (17)		0.85 (24)	
Yes	0.72 (616)		0.70 (143)		0.68 (174)		0.76 (299)	
*Difficulty: Housework*		17.86***		12.76***		6.32*		8.12**
No	0.80 (133)		0.78 (83)		0.81 (23)		0.86 (27)	
Yes	0.72 (586)		0.67 (122)		0.68 (168)		0.76 (296)	
*Difficulty: Paperwork/bills*		9.31**		1.61		1.01		10.02**
No	0.76 (252)		0.74 (76)		0.72 (67)		0.81 (109)	
Yes	0.71 (467)		0.70 (129)		0.68 (124)		0.74 (214)	
*Home and local environment*								
*Home design*		77.57***		24.10***		20.74***		29.85***
Meets needs well	0.81 (359)		0.80 (101)		0.79 (89)		0.82 (169)	
Meets most needs	0.71 (217)		0.69 (66)		0.66 (47)		0.76 (104)	
Meets some needs or totally inappropriate^a^	0.57 (143)		0.55 (38)		0.56 (55)		0.61 (50)	
*Getting around local area*		53.62***		21.39***		15.19***		27.80***
Get to all places	0.83 (225)		0.81 (91)		0.82 (49)		0.85 (85)	
At times find it difficult to get to places	0.74 (252)		0.67 (64)		0.70 (78)		0.80 (110)	
Unable to get to all places or not leave home^a^	0.64 (241)		0.60 (50)		0.59 (64)		0.68 (127)	
*Social contact and loneliness*								
*How often do you lack companionship?*		70.56***		12.11***		27.93***		28.90***
Hardly ever	0.81 (326)		0.80 (71)		0.78 (77)		0.83 (178)	
Some of the time	0.72 (235)		0.70 (77)		0.73 (63)		0.72 (95)	
Often	0.59 (157)		0.62 (57)		0.51 (50)		0.63 (50)	
*How often do you feel left out?*		121.05***		39.99***		29.14***		40.81***
Hardly ever	0.82 (321)		0.83 (75)		0.81 (60)		0.83 (186)	
Some of the time	0.73 (233)		0.74 (66)		0.72 (75)		0.73 (92)	
Often	0.55 (159)		0.55 (62)		0.52 (55)		0.58 (42)	
*How often do you feel isolated from others?*		138.10***		32.07***		41.22***		56.09***
Hardly ever	0.83 (310)		0.83 (68)		0.82 (58)		0.84 (184)	
Some of the time	0.73 (232)		0.73 (71)		0.74 (74)		0.73 (87)	
Often	0.55 (176)		0.58 (66)		0.50 (58)		0.58 (52)	
*Satisfaction with social care services*								
*Satisfaction with services*		50.11***		7.33***		14.87***		29.67***
^a^Extremely, very or quite satisfied	0.77 (533)		0.75 (154)		0.74 (130)		0.80 (249)	
Neither satisfied nor dissatisfied	0.66 (96)		0.64 (21)		0.65 (34)		0.68 (41)	
^a^Extremely, very or quite dissatisfied	0.55 (77)		0.59 (23)		0.49 (26)		0.57 (28)	

* *p* < 0.05 ** *p* < 0.01 *** *p* < 0.001

^ *p* < 0.1

^a^Lowest or highest two/three levels are collapsed

The multivariate regression is shown in Table 6. The variance of the residuals was not found to be homogenous using Cook-Weisberg’s test for heteroscedasticity (Χ^2^(1) = 75.3, *p* < 0.01) [45]; therefore, Huber-White standard errors are reported [46, 47]. The OLS regression model also failed the Ramsey RESET test [48] and Pregibon’s link test [49], which indicates possible model misspecification and omitted variable bias. To explore whether another functional form would improve the fit, we used a beta regression model [50, 51]. This has been proposed as a way of modelling quality of life data, which are typically characterised by a skewed distribution, spikes at the upper or lower bounds, and heteroscedasticity [50, 52]. The results of this analysis were similar to those from the OLS and post-hoc tests do not indicate improvements in explanatory power, so are not reported here.

**Table 6:** Multivariate regression (OLS)

	ASCOT-SCT4 Index	
Variable	Coefficient (B)	Robust Std. Error
Number of I/ADLs with difficulty^a^	−0.008***	0.002
Home design: Meets most needs^b^	−0.034*	0.014
Home design: Meets some needs or inappropriate for needs^b^	−0.115***	0.020
Local environment: At times I find it difficult to get to places^c^	−0.054**	0.016
Local environment: I am unable to get to places or do not leave home^c^	−0.089***	0.018
Three-item loneliness scale	−0.032***	0.004
Satisfaction with services: Neither satisfied nor dissatisfied^d^	−0.049*	0.020
Satisfaction with services: Extremely, quite or very dissatisfied^d^	−0.131***	0.026
Interview by telephonee^e^	0.008	0.015
Age 65 years or older^f^	0.013	0.013
Primary support reason: Mental health^g^	−0.033*	0.017
Constant	1.109***	0.034
Model Statistics		
N		640
F Statistic		36.6***
Adjusted R^2^		0.442

* *p* < 0.05 ** *p* < 0.01 *** *p* < 0.001

^a^ Scale of 13 I/ADLs (getting in and out of bed; washing hands and face; having a bath or shower; dressing or undressing; using the toilet; eating (including cutting up food); taking medicines; getting around indoors; getting up or down stairs; getting out of the house; shopping; routine housework or laundry; and paperwork or paying bills)

^b^ Base category: Meets my needs very well

^c^ Base category: I can get to all places

^d^ Base category: Extremely, very or quite satisfied with services

^e^ Base category: Interview conducted face-to-face

^f^ Base category: 18–64 years

^g^ Base category: Primary support reason of physical disability or sensory impairment

While controlling for other factors, all hypothesised relationships between individual characteristics and ASCOT-SCT4 index were significant at the 5% level. No significant association was observed for administration of the survey by telephone or by age group (*p* > 0.05). After controlling for other characteristics, those who had a primary support reason due to mental health had significantly lower social care-related quality of life compared to people whose support reason was due to physical disability or sensory impairment (B = −0.033, *p* < 0.05).

In the analysis (Fisher’s exact) of the association between individual characteristics and the two revised items in the ASCOT-SCT4 (*Food and drink* and *Dignity)*, *Food and drink* is significantly associated with the ADL of feeding yourself including the ability to cut up food (*p* < 0.001). Those who reported that they could undertake this activity alone without difficulty were more likely to report the ideal state in this attribute. Self-rated suitability of the home environment and the accessibility of the local environment are significantly associated with this attribute, as were also indicators of loneliness and social isolation (*p* < 0.001). As in previous analysis (Malley et al. [9]), the expected association between the rating of this attribute and the IADL of shopping did not reach significance.

Dignity refers to the psychological impact of the social care services on the service user’s sense of personal significance. Significant associations were observed between rating of *Dignity* and satisfaction with services, self-perceived social isolation and loneliness, accessibility of the local environment, and home design (*p* < 0.01). The significant association with home design was not anticipated in the hypotheses for this study (see Table 2); however, it replicates the findings of a study of older adults in England who received home care support [9]. This may reflect an underlying association with compromises in the quality of care delivered due to the unsuitability of the home layout or design. Unexpected significant associations at the 5% level were also found between rating of *Dignity* and three of the ADLs related to personal care (getting in/out of bed; bathing or showering; and getting dressed or undressed), which reflects a greater proportion of the sample who reported difficulty with these personal care tasks rating some or high-level needs in *Dignity*. This may be due to some overlap between the two concepts of the *Dignity* filter question (i.e. the effect of needing help per se) and *Dignity* (i.e. the effect of how you are helped), especially for people who receive support with personal care where even responsive, person-centred care may leave the person feeling undermined.

Associations between indicators of service quality and *Dignity* were considered for the subsample of respondents who received home care services (*n* = 454). *Dignity* was significantly associated with all of the user-reported indicators of home care quality^2^ such that higher quality services were related to higher likelihood of reporting the ideal state for this attribute (*p* < 0.001). Importantly, there was a significant association with the overall rating of how the respondent felt s/he was treated by care worker(s) and *Dignity;* those who reported that they were not always happy with how they were treated were more likely to report high-level needs and less likely to report the ideal state*.*

### Test-retest reliability

In the test-retest analysis, all items demonstrated fair to substantial test-retest reliability (κ = 0.27–0.72) [40]. The lowest Kappa is calculated for the attribute of *Personal comfort and cleanliness*. The percentage agreement between test and retest scores in this attribute is 68.7%, which is adequate [43]. The most frequent change in response between the initial and follow-up interview was from the ideal state to no needs, or vice versa (25.3%). The preference weights for *Personal comfort and cleanliness* indicate that the perceived difference between these outcome states is smaller than between other levels [13]. Therefore, it is anticipated that any change from the ideal state to no needs, or vice versa, will not affect the reliability of the utility-weighted ASCOT-SCT4 index score as significantly as would changes between other states. The ICC for the ASCOT-SCT4 index score indicates good test-retest reliability (ICC = 0.783; 95% CI: 0.678–0.857).

## Discussion

While other studies have established the construct validity and suitability of the English version ASCOT-SCT4 for use with older adults [9, 20], this study has established its construct validity and test-retest reliability with adults, aged 18 years or older, with physical, sensory or mental health-related support needs who use community-based social care services in England. In summary, the analysis presented in this paper provides support for the construct validity of the overall ASCOT-SCT4 index as a measure of social care-related quality of life.

In the absence of a ‘gold standard’ measure of social care-related quality of life, we considered convergent validity with instruments that measure related constructs. As hypothesised, the ASCOT-SCT4 and ICECAP-O/A or overall QoL are more strongly positively correlated than the ASCOT-SCT4 and EQ-5D-3 L. This supports the notion that there is conceptual overlap between the ASCOT construct of SCRQoL and ICECAP’s ‘capability wellbeing’. Indeed, some of the ASCOT and ICECAP-O attributes are very similar (i.e., control, enjoyment and role), although the phrasing of ICECAP-O focuses on the ability to achieve broad aspects of QoL [53] while ASCOT focuses on both functioning and capability in relation to aspects of QoL that may be influenced by social care [13]. The moderate correlation with EQ-5D-3 L is consistent with SCRQoL as a broader construct than HRQoL, with some overlap between these constructs due to the definition of ASCOT-SCT4’s lowest level of outcome as high-level social care needs that may affect health [13].

The construct validity findings are consistent with an earlier study of older adults in England who used home care services, which found a stronger positive relationship between SCRQoL and measures of wellbeing, control and autonomy than for health-related quality of life [9, 13]. The analysis presented in this article is also consistent with a study of community-dwelling older adults in Australia, in which the correlation between the ASCOT-SCT4 and another measure of quality of life, the OPQoL-Brief [54], was found to be stronger than with the EQ-5D-3 L [6, 20]. Similarly, in a sample of older adults in the Netherlands, the correlation between the ICECAP-O and the cross-culturally validated Dutch translation of the ASCOT-SCT4 was found to be stronger than between the ASCOT-SCT4 and the EQ-5D-3 L [8, 21]. The relationship between the ASCOT-SCT4 and the individual EQ-5D-3 L item scores in this study, with stronger correlations between SCRQoL and the EQ-5D-3 L attributes of usual activities and anxiety/depression, are also consistent with previous study of older adults who use day care services in England [5]. In this study we have begun to explore the relationships between the ASCOT-SCT4, EQ-5D-3 L and ICECAP-A or -O, which expands on this previous research [5, 8, 20, 55], however, given the interest in these measures this is also clearly a direction for future research.

An earlier study identified potential issues with the response distribution and construct validity of *Food and drink* and *Dignity* [9]. The analysis in this paper sought to replicate the construct validity analysis presented in Malley et al. [9] for these two items to establish the construct validity of the revised items. In this study, the response distribution for *Food and drink* is less negatively skewed, which supports the re-wording of the question proposed by [9]. The evaluation of the expected relationships with individual characteristics confirmed the construct validity of the revised item. This study also provides evidence that supports the construct validity of the revised question for *Dignity*. The rating of *Dignity* was found to be related to a number of indicators of the experience and perceived quality of home care, and also to the individual’s need for personal care support in terms of washing, dressing and getting into or out of bed. These findings suggest that the revised item captures the effect of the perceived quality of formal social care on the individual’s personal sense of significance [13]. Further work exploring whether the relationship between rating of *Dignity* and personal care tasks reflects the intended construct of the effect of the *way in which the care is provided* on the individual’s sense of personal significance, rather the *effect of needing help* with personal care regardless of the level of social care need, would be valuable.

This study also indicates that the ASCOT-SCT4 is a feasible instrument, with low levels of non-response for all items (<1%) except for *Dignity* (<4%). The higher percentage of missing data for *Dignity* may be explained by the inapplicability of the revised item for respondents who received services (for example, equipment or household adaptations) that do not involve ongoing, regular interaction with care professionals. Of the cases of missing data (*n* = 25), twenty cases represent respondents who reported using equipment/adaptations without any other type of ongoing social care support. This should be considered in future use of the questionnaire with a diverse sample, in which respondents may receive interventions that do not involve personal interaction with care workers. Although *Dignity* was developed in the English context to capture the positive or negative effect on an individual’s sense-of-self due to the way in which care and support are delivered [13], a qualitative study of older South Australians found that respondents understood the item to also refer to unpaid care by friends or relatives as well as wider community support [6]. Therefore, although the revised item may focus the respondent on the intended construct, it clearly makes the instrument less suitable for samples where respondents are not in receipt of social care delivered through face-to-face contact with paid care staff.

In terms of test-retest reliability of the ASCOT-SCT4, this study indicates that the ASCOT-SCT4 index has good reliability. This is consistent with the finding of a study of older adults in the Netherlands who completed the Dutch translation of the ASCOT-SCT4, which found good test-retest reliability for all the items [8]. Notably, however, unlike the Dutch study, the *Personal comfort and cleanliness* item has low stability albeit that the percentage agreement is acceptable. A significant association between the rating of this attribute and self-rated change in social care need (I/ADLs) between baseline and follow-up indicates that the item instability may be, at least partly, attributable to fluctuations in care needs. It could be argued that the comparative ‘unreliability’ of this item demonstrates the validity of the measure, since it is sensitive to fluctuations in conditions that contribute to quality of life.

While the findings of this study contribute to the evidence for the construct validity, test-retest reliability and feasibility of the ASCOT-SCT4 measure in a diverse sample of users of social care services, there are some limitations to this study. First, the ASCOT-SCT4 questionnaire used in this study erroneously omitted the revised wording for the *Accommodation* item (see Appendix [9]). Therefore, further testing of the construct validity and response distribution of the revised item is still required. Second, the test-retest reliability analysis in this study was limited to a subsample of adults with physical disability or sensory impairment. Further research is required to establish test-retest reliability in a wider range of users of social care services. Although the level of missing values is an indicator of feasibility of the ASCOT-SCT4 attributes and response levels, the study only considered data collection by face-to-face or telephone interview. Further research would be required to explore missing data and other indicators of feasibility (e.g. consent, response rates) with data collection by self-completion.

Also, the study sample was limited only to users of social care services with physical disabilities, sensory impairment or mental health condition and who had the capacity to participate in a structured interview. These study criteria excluded users of social care with learning disabilities, as well as people who may lack capacity to complete the standard version of the questionnaire but may be able to express their views with additional support, communication aids, or using different methodology. In order to be able to evaluate social care services for wider groups of users, it is necessary to develop approaches to establishing SCRQoL outcomes: for example, the ASCOT includes a method for care homes [56], an easy-read self-completion format for adults with intellectual disabilities [57] and a proxy version in development. As the number of ASCOT measures proliferates to address the diverse support and access needs of social care users, a method for establishing read-across between measures is needed to compare the effectiveness of services for different groups of social care users.

## Conclusions

This study indicates that the ASCOT-SCT4 is a valid and feasible measure of social care-related quality of life in adults who use social care services, which supports its use in studies that seek to capture social care outcomes. Further research would be of value to establish the instrument’s test-retest reliability with a more diverse sample of social care users, the feasibility of data collection by self-completion, further comparison of the ASCOT-SCT4 to other measures (e.g. ICECAP, EQ-5D), and the validity, reliability and comparability with adapted versions of the ASCOT (for example, the Easy-Read version).

## Appendix

### Revised wording of the ASCOT-SCT4 questionnaire

In this study, the following prompt was included after the *Dignity* question: When you are thinking about the way you are helped and treated, please think about the help you get from *[Formal carers].* Do not include the help you get from *[Informal carers].*

## Abbreviations

ADLs: Activities of daily living
ASCOF: Adult Social Care Outcomes Framework
ASCOT: Adult Social Care Outcomes Toolkit
ASCS: Adult Social Care Survey
EQ-5D-3 L: EuroQoL 5-item with 3 levels of response questionnaire
IADLs: Instrumental activities of daily living
ICC: Intra-class correlation coefficient
ICECAP-A: Investigating Choice Experiments for the preferences of older people Capability measure for adults
ICECAP-O: Investigating Choice Experiments for the preferences of older people Capability measure for older people
LA: Local authority
MH: Mental health
OLS: Ordinary least squares
PDSI: Physical disability or sensory impairment
QoL: Quality of life
SCRQoL: Social care-related quality of life
SCT4: Four-level self-completion questionnaire

## Footnotes

1 By comparison to the other outcome measures considered in this study, the percentage of missing data was also <1% for all EQ-5D and ICECAP-A items. Three of the ICECAP-O items also had a missing data rate of <1%. The two items with the highest percentage of missing data were attachment (4 (1.3%)) and security (7 (2.3%)).

2 The analysis included the following indicators of home care quality: whether care workers come at times that suit you; whether care workers do what you want done; whether care workers are in a rush; whether care workers spend less time per visit than they are supposed to; whether you are kept informed of changes to the home care service; whether you are happy with how care workers treat you; and the quality of the relationship with care worker(s).
